# 

**Published:** 1820-09-01

**Authors:** 


### Contents.

No. 2, SEPTEMBER, 1820.

ART. I.

PERITONEAL INFLAMMATION.

1. M. BROUSSAIS on Inflammation of the Peritoneum - 161

2. M. GASC on Peritonitis - - - - - - - - - - - - - 161

3. Dr. ABERCROMBIE on Enteritis - - - - - - - - - - 161

4. Dr. PEMBERTON on the Abdominal Viscera - - - 161

5. MONTFALCON on the Peritoneum - - - - - - - - 161

II.

Dr. MARSHALL HALL on a serious Morbid Affection, chiefly occurring after Delivery, Miscarriage, &c. - - - - 195

III.

Letters to the President of the Associated Apothecaries, &c. on the present State of the Practice of Physic and Surgery - - - - - - - - - - - - - - - - - 204

IV.

Mr. W. WHITE on Strictures of the Rectum, and other Affections, which diminish the Capacity of that Intestine; with Cases and Engravings - - - - - - - - - - - 208

V.

Mr. HOWSHIP on Diseases of the lower Intestines and Anus, &c. &c. &c. - - - - - - - - - - - - - - - - - 222

VI.

An Essay on lessening Pain and facilitating difficult Parturition. By Dr. W. P. DEWEES - - - - - - - - - - - 229

VII.

INSANITY.

1. Dr. HASLAM on Sound Mind - - - - - - - - - - - 237

2. Dr. BURROWS on certain Errors relating to Insanity - - 237

3. Dr. ESQUIROL on Insanity - - - - - - - - - - - - 237

VIII.

Mr. CHARLES BELL's Treatise on the Urethra, Vesica Urinaria, Prostate, and Rectum, with Notes by Mr. JOHN SHAW, Demonstrator of Anatomy, &c. &c. - - - 264

IX.

Dr. HENNEN's Principles of Military Surgery, (3d Edition) exhibiting a View of the Syphilitic Question, and important Documents illustrative of the Mercurial and Non-mercurial Modes of Treatment comparatively - - 283

X.

Mr. CROSS on the Varioloid Epidemic of Norwich, with a General Exposition of Varioloid Diseases - - - - 300

MISCELLANEOUS INTELLIGENCE - - - - - - - - 318—321

Regulations of the Associated Apothecaries, with Remarks 321
Dr. Lucas on the Circulation - - - - - - - - - - - - 329
Lectures, Works in the Press, &c. - - - - - - - - 334

